# Parthenolide leads to proteomic differences in thyroid cancer cells and promotes apoptosis

**DOI:** 10.1186/s12906-022-03579-0

**Published:** 2022-04-02

**Authors:** Meng Cui, Zhe Wang, Le-Tian Huang, Jia-He Wang

**Affiliations:** 1 Department of Oncology, Shengjing Hospital of China Medical University, Shenyang, 110004 Liaoning China; 2 Department of Hospice Care, Shengjing Hospital of China Medical University, Shenyang, 110004 Liaoning China; 3 Department of General Medical, The Second Affiliated Hospital of Medical College, Xian Jiaotong University, Xian, 710004 Shaanxi China; 4 Department of Family Medicine, Shengjing Hospital of China Medical University, No.36 Sanhao Road, Heping District, Shenyang City, 110004 Liaoning Province People’s Republic of China

**Keywords:** Parthenolide, Thyroid cancer, Proteomic, Apoptosis, BCPAP

## Abstract

**Background:**

Parthenolide has anti-inflammatory, immunomodulatory and anti-cancer activities. But its effect on thyroid cancer cells is still largely unknown.

**Methods:**

Label-free quantitative proteomics and bioinformatics analysis were used to investigate the differentially expressed proteins and their functions in thyroid cancer treated with parthenolide and control pair. Hoechst 33258 fluorescent staining and Annexin V-FITC/PI double staining flow cytometry were used to detected BCPAP cells apoptosis. Parallel reaction monitoring (PRM) and quantitative real-time PCR were used to verify the expression of apoptosis-related differential proteins and their mRNA.

**Results:**

Sixty up-regulated and 96 down-regulated differentially expressed proteins were identified in parthenolide treated thyroid cancer cells BCPAP compared with control thyroid cancer cells. The proteins were mainly relevant to various biological processes that included metabolic processes, response to extracellular stimulus and interaction with host. The molecular functions of most differentially expressed proteins were associated with binding functions and nucleotidyltransferase activity. According to the Kyoto Encyclopedia of Genes and Genomes, the differentially expressed proteins identified are primarily related to various types of metabolic pathways and DNA replication. In cell experiments in vitro, with the increase of the dose of parthenolide, the number of cells gradually decreased, the apoptosis rate gradually increased. PRM verified that the apoptosis-related proteins HMOX1 and GCLM were up-regulated and IL1B was down-regulated in BCPAP cells treated with parthenolide. The mRNA expressions of HMOX1, GCLM, ITGA6 and CASP8 were up-regulated and HSPA1A was down-regulated by PCR.

**Conclusions:**

Parthenolide may influence the biological behavior of human thyroid cancer cells by affecting the expression of proteins related to cell metabolism and DNA replication. Parthenolide induced significant cellular morphological changes and apoptosis in human thyroid cancer cells, leading to an anti-proliferative effect.

## Background

The incidence of thyroid carcinoma has increased dramatically in the past few decades due to improvement in diagnostic accuracy [1]. More than 56,000 new thyroid cancer cases were diagnosed in the United States in 2017, accounting for 3.4% of all new cancers [2]. Thyroid cancer has become one of the most rapidly occurring tumors and the most common endocrine malignant tumors. Papillary carcinoma is the most common histological type, accounting for approximately 80% [3]. At present, there are many methods in the treatment of well-differentiated thyroid cancer such as surgical resection, radioactive iodine, thyroid hormone suppression therapy [4], and so on. However, with the decrease of thyroid cancer differentiation, traditional surgery and radioactive iodine ablation no longer have a good effect on partially differentiated thyroid [5]. Therefore, in order to provide more options for thyroid cancer treatment, new therapeutic drugs and treatment methods have become one of the research hotspots in the medical field [6, 7].

Natural or herbal extracts have anticancer effects in different cancers, and can also be used be combined with traditional chemotherapy drugs to enhance their anticancer functions [8, 9]. A recent research confirmed that Dihydrotanshinone exerts antitumor effects on anaplastic thyroid cancer cells by reducing MAD2 expression levels and has a synergistic effect with cisplati [10]. Parthenolide (PTL) is the principal ingredient of the herb feverfew (*Tanacetum parthenium L. Schulz Bip.*), which has a variety of biological activities, including antibacterial, anti-inflammatory, neuroprotection, immune regulation and anti-cancer. It is a traditional herb widely used in Asian countries and has been used for many years to treat fever, headache and arthritis [11, 12].

Previous studies have shown that PTL can inhibit proliferation and induce apoptosis of various tumor cells such as liver cancer, gastric cancer, pancreatic cancer and so on [13–17]. Although the anticancer effect of PTL has been demonstrated in several cancer cells, its anticancer potential and mechanism in human thyroid cancer BCPAP cells have not been fully elucidated.

Proteomics has only recently matured and is now capable of accurate quantitative analysis of most expressed proteins across hundreds of samples [18]. Proteins are the executors of the functional coding of the cell genome, differences in protein expression under different conditions may be closely related to the biological functions of cancer cells. Therefore, identifying the differences in protein expression in BCPAP cells under the effect of PTL is helpful to identify the function of PTL.

In the present study, proteomic differences of thyroid cancer cells under the effect of PTL were explored, and the pro-apoptotic effect of PTL on thyroid cancer cells was investigated in vitro.

## Methods

### Materials

Human papillary thyroid carcinoma cell line BCPAP was purchased from Shanghai Institutes for Biological Sciences, China. PTL was purchased from Absin (Shanghai, China). RPMI 1640 medium was purchased from Corning, USA. Fetal bovine serum was purchased from Shuangru Biology Science&Technology Co.Ltd. Sodium deoxycholate (SDC), formic acid (FA), ammonium bicarbonate (ABC), TCEP, iodoacetamide (IAA) and rifluoroacetic acid (TFA) were purchased from Sigma-Aldrich, USA. Hoechest 33,258 were purchased from Beyotime, Shanghai, China. Acetonitrile (ACN) was purchased from J.T.Baker, USA. Pierce BCA Protein Assay Kit, Q Exactive and EASY-nLC1200 were purchased from Thermo Fisher Scientific. TRIzol was purchased from Invitrogen life technologies, USA. RNAse Inhibitor was purchased from Enzymatics, USA. SuperScriptTM III Reverse Transcriptase kit was purchased from Invitrogen, USA. 2X PCR master mix was from Arraystar, USA.

### Cell culture and drug treatment

BCPAP was maintained in a complete RPMI 1640 medium, in which 10% fetal bovine serum, 100 U/mL penicillin, 100 U/mL streptomycin were added. Cells were cultured in an environment of 5% CO_2_, 37 °C. Sufficient cell samples were divided into 6 groups for further proteomic analysis. According to the appropriate concentration (IC50) explored in the preliminary experiment, PTL was dissolved in 0.05% DMSO and diluted with PBS to 10 μmol/L. PTL was then added to 3 treatment groups for 24 h, and 3 control groups were added with the same amount of DMSO and PBS for the same time. Label-free quantitative proteomics and parallel reaction monitoring (PRM) were grouped and processed in the same way.

### Protein preparation

Radio Immunoprecipitation Assay (RIPA) buffer was configured with 25 mmol/L Tris-HCl with the pH 7.6, 150 mmol/L NaCl, 1% SDS, 1% sodium deoxycholate and 1% NP-40. It was mixed with protease inhibitor and 1 mmol/L Phenylmethanesulfonyl fluoride (PMSF) before use and chilled. Sample (100 mg, 1 × 10^7^cell) was added in 1000 μL RIPA buffer, dissolved at 4 °C, then centrifuge 15 min with top speed. The supernatant was collected for protein detection and quantification using a Pierce BCA Protein Assay Kit. For each sample, 100 μg proteins were took and diluted to 1 mg/ml. Acetone was pre-chilled to − 20 °C, 4-6 fold volumes of acetone was added into the sample, shaken on the ice for 30 min. Supernatant was discarded after centrifugation at top speed at 4 °C. The precipitation was washed twice with 200 μL 80% chilled acetone.

### Protein enzymolysis

Ammonium bicarbonate 200 μL (100 mmol/L with 1% SDC) were added in, mixed in vortex and simply centrifuged. The protein precipitation was fully dissolved by water bath ultrasound for 5-30 min. TCEP was added to each sample to 5 mmol/L and incubated for 10 min at 55 °C. After each sample was cooled to room temperature (RT), iodoacetamide was added to 10 mmol/L and incubated in dark for 15 min. The resuspension buffer was used to dissolve trypsin to 0.5 μg/μL, left for 5 min at RT, then added to each sample at a ratio of protein: trypsin = 50: 1. The resultant samples were incubated at 37 °C with thermomixer overnight. TFA was added to mixed sample (final concentration 2%, pH < 2) to precipitate SDC. Centrifuged at top speed, and then the supernatants were transferred to new tubes. N × 100 μL 2% TFA was added and thoroughly mixed. The step was repeated twice. Three supernatants were merged and centrifuged at top speed for 15 min. The supernatants were then desalted by a C_18_ extraction column.

### LC-MS/MS analysis

The nano-UPLC liquid phase system EASY-nLC1200 was used to separate and the Q-Exactive mass spectrometry was used to detected 2 μg polypeptides from each sample. Analysis was carried out by a reversed-phase column, the mobile phase A was 0.1% formic acid ACN solution (ACN 2%), the phase B was 0.1% formic acid ACN solution (ACN 80%). Mobile phase A was used for sample separation. For label-free quantitative proteomics, the flow rate was 300 nL/min and the gradient length was 240 min. Phase B: 8 to 35% for 212 min, 35 to 45% for 20 min, 45 to 100% for 2 min, 100% for 2 min, 100 to 2% for 2 min and 2% for 2 min. For PRM, the flow rate was 300 nL/min and the gradient length was 120 min. Phase B: 6 to 28% for 92 min, 28 to 40% for 20 min, 40 to 100% for 2 min, 100% for 2 min, 100 to 2% for 2 min and 2% for 2 min.

Data was collected in positive mode. The top 20 most intense ions were broken up after each full scan. The resolution of MS1 was 70,000 at m/z 200 and of MS2 was set to 17,500. Automatic gain control target of MS1 was set to 3.0E^+ 6^, while of MS2 was set to 1.0E^+ 5^. For label-free quantitative proteomics, the maximum IT for MS1 was 50 ms, 45 ms for MS2. For PRM, the Max IT was 200 ms. The normalized collision energy was 28%, the isolation window was 2 m/z, and the dynamic exclusion time window was 40 s.

### Label-free quantitative proteomics data analysis

Primitive. raw files were handled by MaxQuant (Version 1.5.6.0). Protein sequence was downloaded from the UNIPROT database (Uniprot_human_2016_09) [19]. The quantitative type was label-free quantification containing match between run. Trypsin was designated as a specific endonuclease with a maximum of 3 missing sites. Acetyl[protein N-term] and Oxidation[M] were used as variable modifications, Carbamidomethyl[C] as fixed modification, and the maximum variable modification number was 3. The false discovery rate should be less than 0.01. The unique polypeptides without variable modification were used for quantitative purposes.

### PRM data analysis

PRM data analysis was performed using the Skyline software. Transition parameter Settings: parent ions were set to + 2 and + 3 valence, fragment ions were set to + 1 and + 2 valence, B and Y ions were selected for chromatographic peak extraction and quantification. Then, the final quantitative transition was manually screened according to the peak pattern, intensity and SNR of XIC. Method match tolerance: 0.005 m/z; the resolution of MS2 was 17,500 at m/z 200.

### Hoechest 33258 fluorescence staining

According to preliminary experiment, we confirmed the effect of PTL on the proliferation of BCPAP cells, the quantity and activity of BCPAP cells decreased with the increasing of PTL concentration from 8 μmol/L to 12 μmol/L after 24 h. So BCPAP cells were seeded in 6-well plates with 2 × 10^5^ cells in each well, incubated overnight until the cells were adherent. The cells were treated with PTL (dissolved in 0.05% DMSO and diluted with PBS) for 24 h at different concentrations (8, 10, 12 μmol/L). Control group was treated with the same amount of DMSO and PBS for the same time. Cells were fixed with 4% paraformaldehyde for 10 min, washed with ice PBS for 3 times, stained with Hoechest 33258 at RT for 5 min, and washed with PBS again for 3 times. The cell slide was placed on a slide of 30 μL anti-fluorescence quench solution. Images were observed under Nikon E800 fluorescence microscope.

### Flow Cytometry analysis of Annexin V-FITC/PI staining

BCPAP cells were seeded in 6-well plates at 2 × 10^5^ cells in each well, incubated overnight until the cells were adherent. The cells were treated with PTL (dissolved in 0.05% DMSO and diluted with PBS) for 24 h at different concentrations (8, 10, 12 μmol/L). Control group was treated with the same amount of DMSO and PBS for the same time. After drug treatment, cells were harvested into different tubes. After centrifugation at 800 rpm for 5 min, the supernatant was discarded, and the cells were washed twice with ice-cold PBS. Cell suspension was prepared with 100 μL Binding Buffer. Annexin V-FITC (5 μL) and PI (5 μL) were added in each tube, cells were gently shaken evenly and incubated at RT in dark place for 15 min. Flow cytometry was performed within 1 h.

### Quantitative real-time PCR

Cell treatment were consistent with those described above in ‘Cell culture and drug treatment’. RNA in the experiments were obtained from these BCPAP cells treated with or without PTL, which was extracted with Trizol reagent based on the manufacturer’s protocol. Reverse transcription reaction was completed using SuperScriptTM III Reverse Transcriptase. The qPCR was cycled with the ViiA 7 Real-time PCR System (Applied Biosystems) adopting 2X PCR master mix. The amplification efficiency was evaluated by the standard curve. These experiments was repeated three times. The results were analyzed by standard curve method, GAPDH was used as internal reference.

The real-time PCRs required the following primers:

- GAPDH-F: 5′ GGGAAACTGTGGCGTGAT 3′
- GAPDH-R: 5′ GAGTGGGTGTCGCTGTTGA 3′
- Product Length: 299 bp, Ta = 60 °C.
- IL1B-F: 5′ CCGACCACCACTACAGCAAG 3′
- IL1B-R: 5′ TGGACCAGACATCACCAAGC 3′
- Product Length: 260 bp, Ta = 60 °C.
- GCLM-F: 5′ GATGGAGTTAATCTTTCCTTGG 3′
- GCLM-R: 5′ GGTTACTATTTGGTTTTACCTGTG 3′
- Product Length: 160 bp, Ta = 60 °C.
- HMOX1-F: 5′ GCAGAGGGTGATAGAAGAGG 3′
- HMOX1-R: 5′ GTAAGGACCCATCGGAGAA 3′
- Product Length: 231 bp, Ta = 60 °C.
- ITGA6-F: 5′ TGCGTCCCATTCCCATAAC 3′
- ITGA6-R: 5′ ACATTGTCGTCTCCACATCCC 3′
- Product Length: 163 bp, Ta = 60 °C.
- CASP8-F: 5′ CAAATGCAAACTGGATGATGA 3′
- CASP8-R: 5′ GGATGTCCAACTTTCCTTCTC 3′
- Product Length: 92 bp, Ta = 60 °C.
- HSPA1A-F: 5′ CCACCATTGAGGAGGTAGATTAG 3′
- HSPA1A-R: 5′ CTGCATGTAGAAACCGGAAAA 3′
- Product Length: 176 bp, Ta = 60 °C.

### Statistical and bioinformatics analysis

The standardized quantitative results were then statistically analyzed to obtain the corresponding differentiate expressed proteins. There were 5 biological replicates in this experiment, and t-test statistical test was performed. Protein with ratio A/B > 1.5, *P* value< 0.05, unique peptide≥2 was defined as significantly different. Then GO [20] and KEGG [21] databases were used to identify proteins and analyze pathways.

The data were represented as the means±SD of at least three separate independent experiments. SPSS 22.0 software was used for statistical analysis. T-test was used to compare the data differences between two groups, and analysis of variance(ANOVA) was used to compare the data differences among multiple groups. *P* < 0.05 was considered statistically significant.

## Results

### Differentially expressed proteins (DEPs) between the PTL treated group and controlled group

**Fig. 1:**
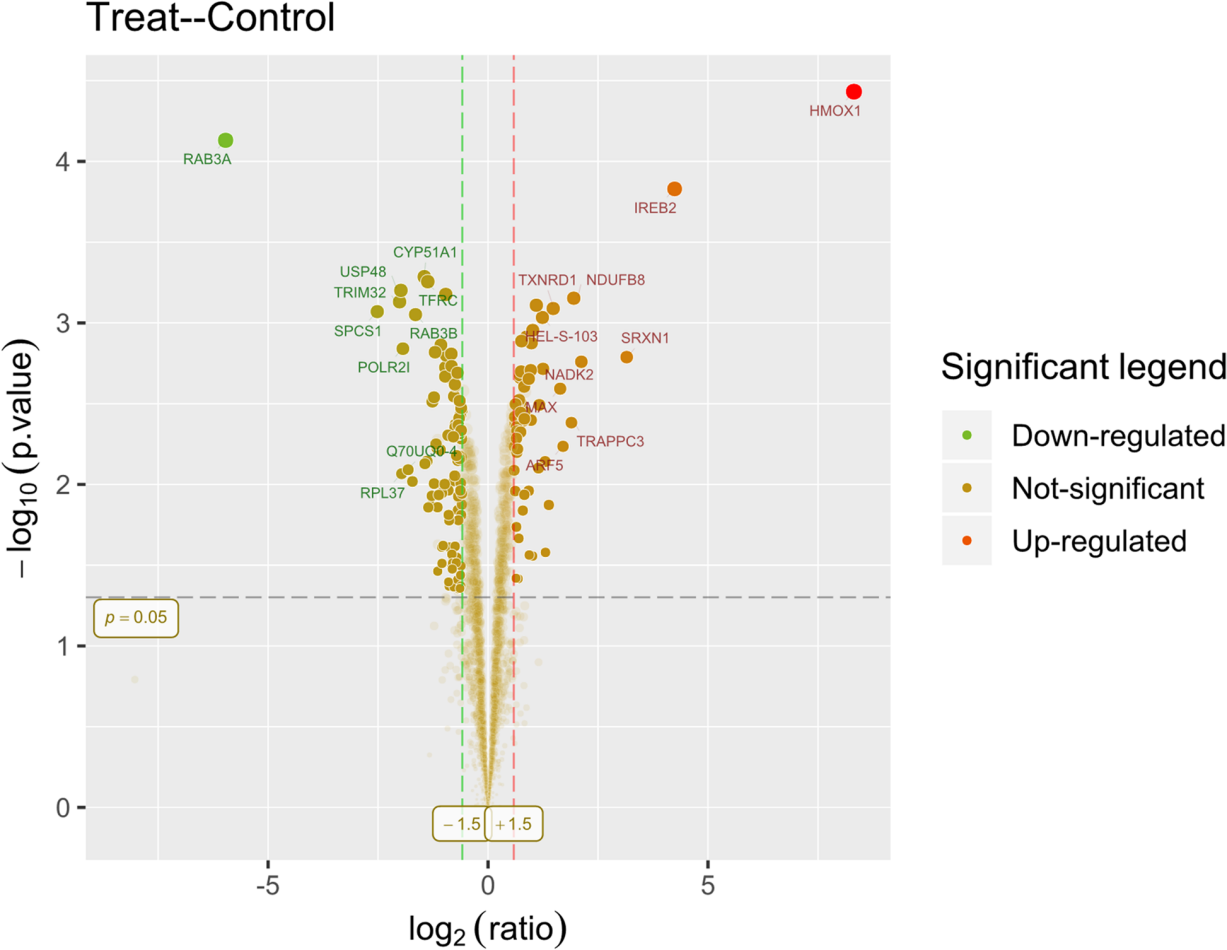
Volcano plot showing of proteomics data. The red/green colors indicate points-of-interest that display both large magnitude fold-changes (x axis) and high statistical significance (−log10 of *P* value, y axis). The *p*-value cutoff was shown by the dashed horizontal line, and the down/up regulated proteins were indicated by the two vertical dashed lines. Transparent points in the significant region mean these proteins don’t satisfy some other conditions. (e.g. unique peptides less than 2)

**Fig. 2:**
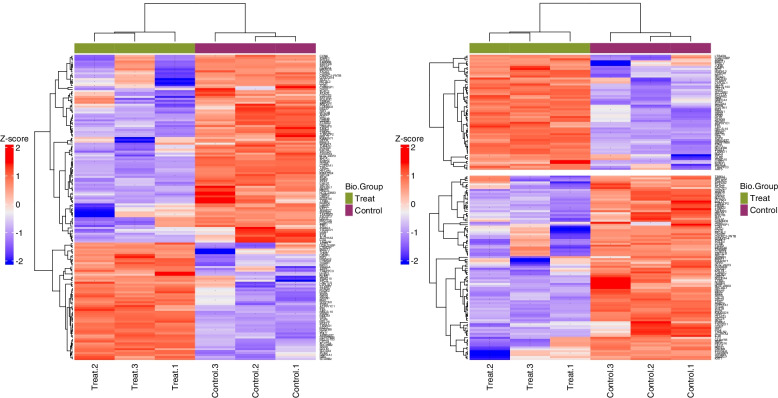
Clustering heatmap of all significant proteins. Pearson’s distance was used if there are 3 or more samples, or Euclidean’s distance if not. Miss values are indicated with‘-’

Compared with the controlled group, 60 up-regulated DEPs and 96 down-regulated DEPs were identified from 2703 quantified proteins in the PTL treated group. A volcano plot was used to show all the DEPs (Fig. 1). The false discovery rate(FDR) of polypeptide and protein level in this experiment was controlled at 0.01. The difference multiple selection identification and quantitative results were as follows: the protein with different expression multiplier(ratio A/B) > 1.5, *P* value< 0.05 was defined as significant difference protein. Significant proteins in Treat-Control group are clustered as heatmap (Fig. 2).

### GO enrichment

According to molecular function, biological processes and cellular component, DEPs were enriched by GO (*P* < 0.05), and the results were shown in Tables 1 and 2.

**Table 1:** Enriched GO terms in molecular functions (*P* < 0.05)

ID	Description	GeneRatio	*P*-value
GO:0048037	cofactor binding	19|149	3.56E-08
GO:0051539	4 iron, 4 sulfur cluster binding	7|149	4.88E-08
GO:0051536	iron-sulfur cluster binding	8|149	6.62E-08
GO:0051540	metal cluster binding	8|149	6.62E-08
GO:0001618	virus receptor activity	7|149	3.09E-06
GO:0104005	hijacked molecular function	7|149	3.09E-06
GO:0008483	transaminase activity	4|149	2.67E-05
GO:0016779	nucleotidyltransferase activity	8|149	2.88E-05
GO:0016769	transferase activity, transferring nitrogenousgroups	4|149	3.90E-05
GO:0030350	iron-responsive element binding	2|149	7.16E-05
GO:0003887	DNA-directed DNA polymerase activity	4|149	0.000100511
GO:0016667	oxidoreductase activity, acting on a sulfur groupof donors	5|149	0.000167427
GO:0004748	ribonucleoside-diphosphate reductase activity, thioredoxin disulfide as acceptor	2|149	0.000213652
GO:0016728	oxidoreductase activity, acting on CH or CH2 groups, disulfide as acceptor	2|149	0.000213652
GO:0061731	ribonucleoside-diphosphate reductase activity	2|149	0.000213652
GO:0047485	protein N-terminus binding	6|149	0.000334432
GO:0016651	oxidoreductase activity, acting on NAD(P)H	6|149	0.000405851
GO:0009055	electron transfer activity	6|149	0.00042544
GO:0034061	DNA polymerase activity	4|149	0.000434811
GO:0045296	cadherin binding	10|149	0.000454903
GO:0015036	disulfide oxidoreductase activity	4|149	0.000476266
GO:0015038	glutathione disulfide oxidoreductase activity	2|149	0.000704247
GO:0015035	protein disulfide oxidoreductase activity	3|149	0.00120311
GO:0004128	cytochrome-b5 reductase activity, acting onNAD(P)H	2|149	0.001462482
GO:0001882	nucleoside binding	10|149	0.001685683
GO:0017022	myosin binding	4|149	0.002016304
GO:0097718	disordered domain specific binding	3|149	0.002265406

**Table 2:** Enriched GO terms in biological process (*P* < 0.05)

ID	Description	GeneRatio	*P*-value
GO:0051701	interaction with host	12/152	2.92E-07
GO:0015949	nucleobase-containing small moleculeinterconversion	6/152	3.37E-07
GO:0009064	glutamine family amino acid metabolic process	7/152	3.09E-06
GO:0097327	response to antineoplastic agent	7/152	1.35E-05
GO:0006520	cellular amino acid metabolic process	13/152	1.78E-05
GO:1901605	alpha-amino acid metabolic process	10/152	2.12E-05
GO:0009991	response to extracellular stimulus	15/152	2.49E-05
GO:0002705	positive regulation of leukocyte mediatedimmunity	7/152	2.50E-05
GO:0002708	positive regulation of lymphocyte mediatedimmunity	6/152	6.56E-05
GO:0046718	viral entry into host cell	7/152	7.65E-05
GO:0032201	telomere maintenance via semi-conservativereplication	4/152	7.95E-05
GO:0002703	regulation of leukocyte mediated immunity	8/152	0.000104476
GO:0009065	glutamine family amino acid catabolic process	4/152	0.000121625
GO:0002699	positive regulation of immune effector process	8/152	0.000138637
GO:0030260	entry into host cell	7/152	0.000168563
GO:0044409	entry into host	7/152	0.000168563
GO:0051806	entry into cell of other organism involved insymbiotic interaction	7/152	0.000168563
GO:0051828	entry into other organism involved in symbiotic interaction	7/152	0.000168563
GO:0031667	response to nutrient levels	13/152	0.000196234
GO:1901607	alpha-amino acid biosynthetic process	5/152	0.000208321
GO:0031630	regulation of synaptic vesicle fusion topresynaptic active zone membrane	2/152	0.000219716
GO:0009263	deoxyribonucleotide biosynthetic process	3/152	0.000263943
GO:2001237	negative regulation of extrinsic apoptoticsignaling pathway	6/152	0.00026556
GO:0019048	modulation by virus of host morphology orphysiology	4/152	0.00031054
GO:0071496	cellular response to external stimulus	10/152	0.000333104
GO:0009112	nucleobase metabolic process	4/152	0.000417598

**Fig. 3:**
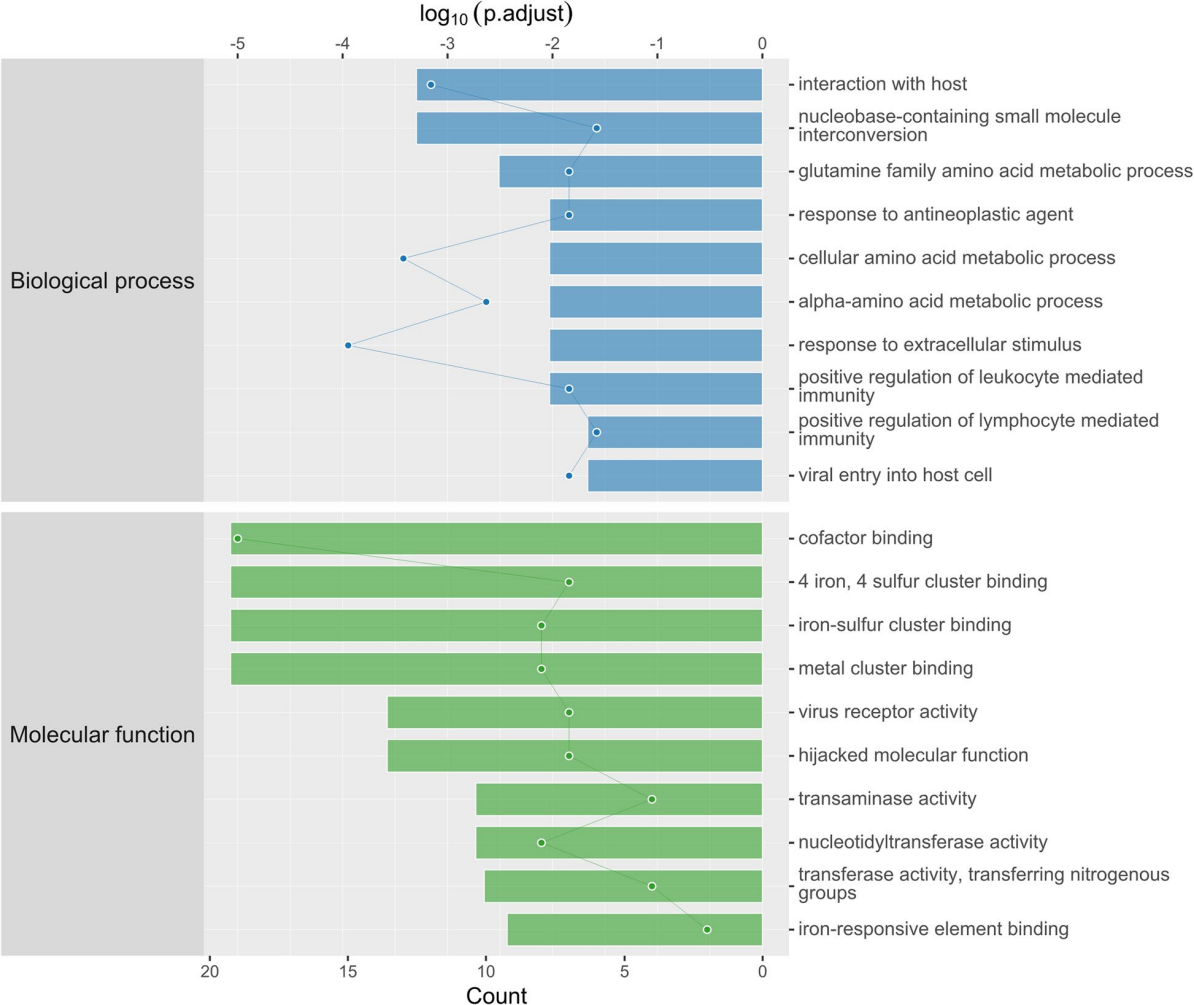
Enriched GO items. Bar: log_10_ (*p*.adjust); Point: Count

When the molecular functions of these DEPs were analyzed, most DEPs were related to binding functions, as shown in Table 1, including ‘cofactor binding’, ‘cadherin binding’ and ‘nucleoside binding’. As shown in Fig. 3, the most significant difference in expression is cofactor binding.

When the biological processes of these DEPs were analyzed, as shown in Table 2 and Fig. 3, the majority and most significant DEPs were associated with ‘response to extracellular stimulus’, ‘interaction with host’ and ‘cellular amino acid metabolic process’.

### KEGG pathway analysis

**Fig. 4:**
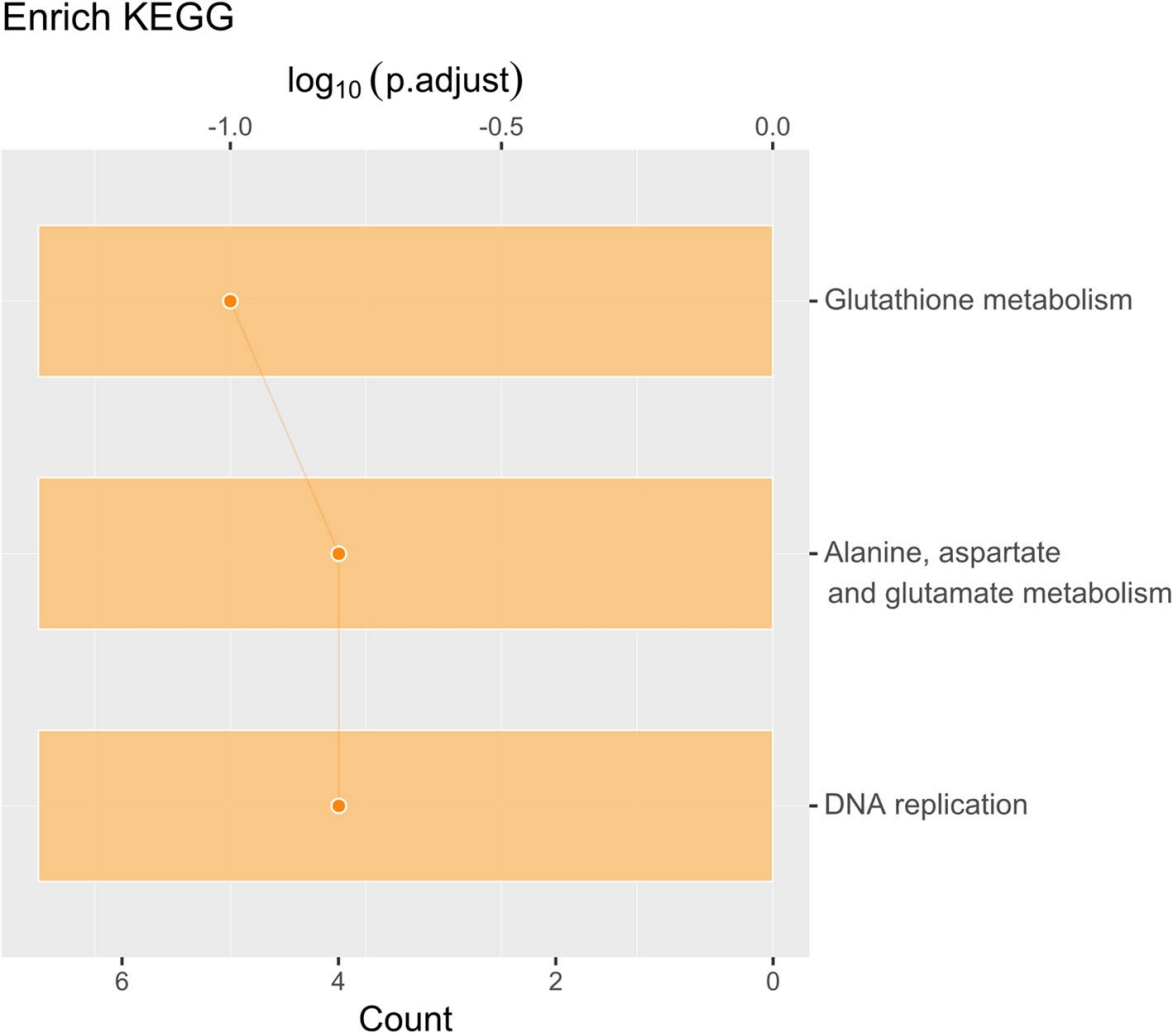
Enriched KEGG items. Bar: log_10_(*p*.adjust); Point: Count

KEGG analysis results, as shown in Fig. 4, showed significant DEPs enrichment in ‘Glutathione metabolism’, ‘Alanine, aspartate and glutamate metabolism’ and ‘DNA replication’ [21].

### Morphological changes of apoptosis

**Fig. 5:**
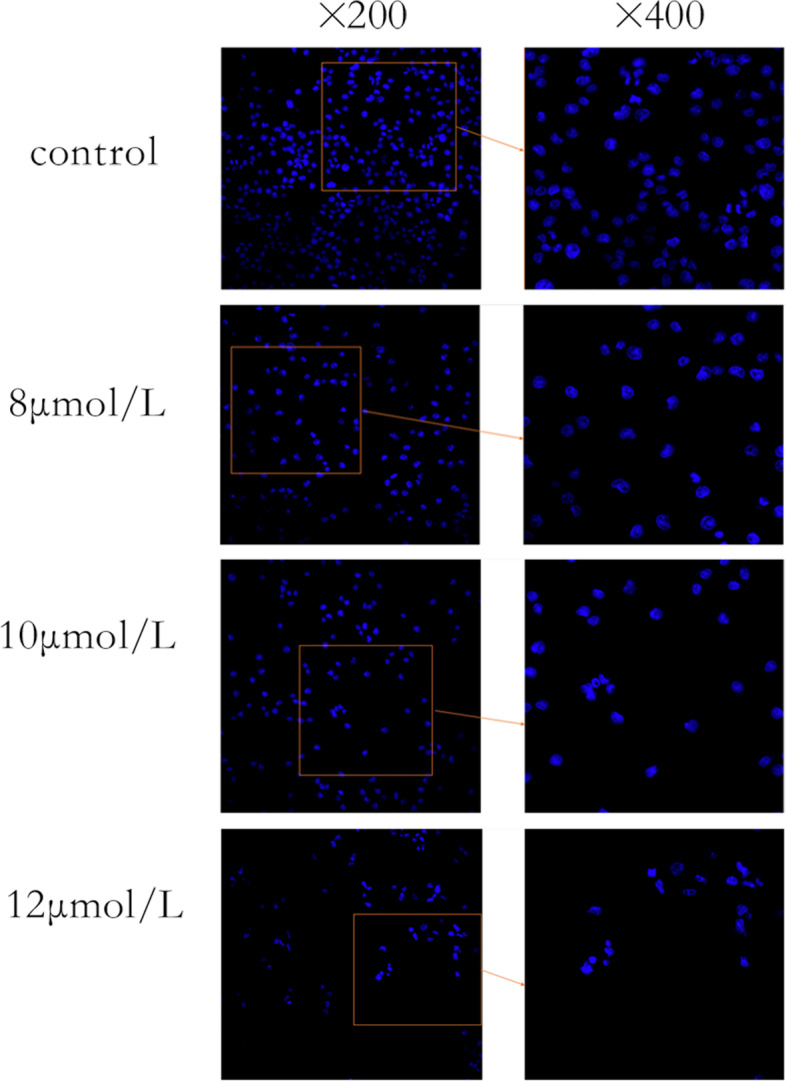
BCPAP cells apoptosis were detected by Hoechst 33258 Staining

BCPAP cells were treated with different concentrations of PTL (8, 10, 12 μmol/L) for 24 h respectively, and the cell morphology was observed after staining with Hoechst 33258. In the control group, there were many cells with high density, regular nuclear morphology, uniform and diffuse blue fluorescence. With the increase of drug concentration, the number of cells decreased gradually, the cell morphology became irregular, the fluorescence became denser, and the normal nuclear morphology was lost (Fig. 5).

### PTL induced BCPAP apoptosis

**Fig. 6:**
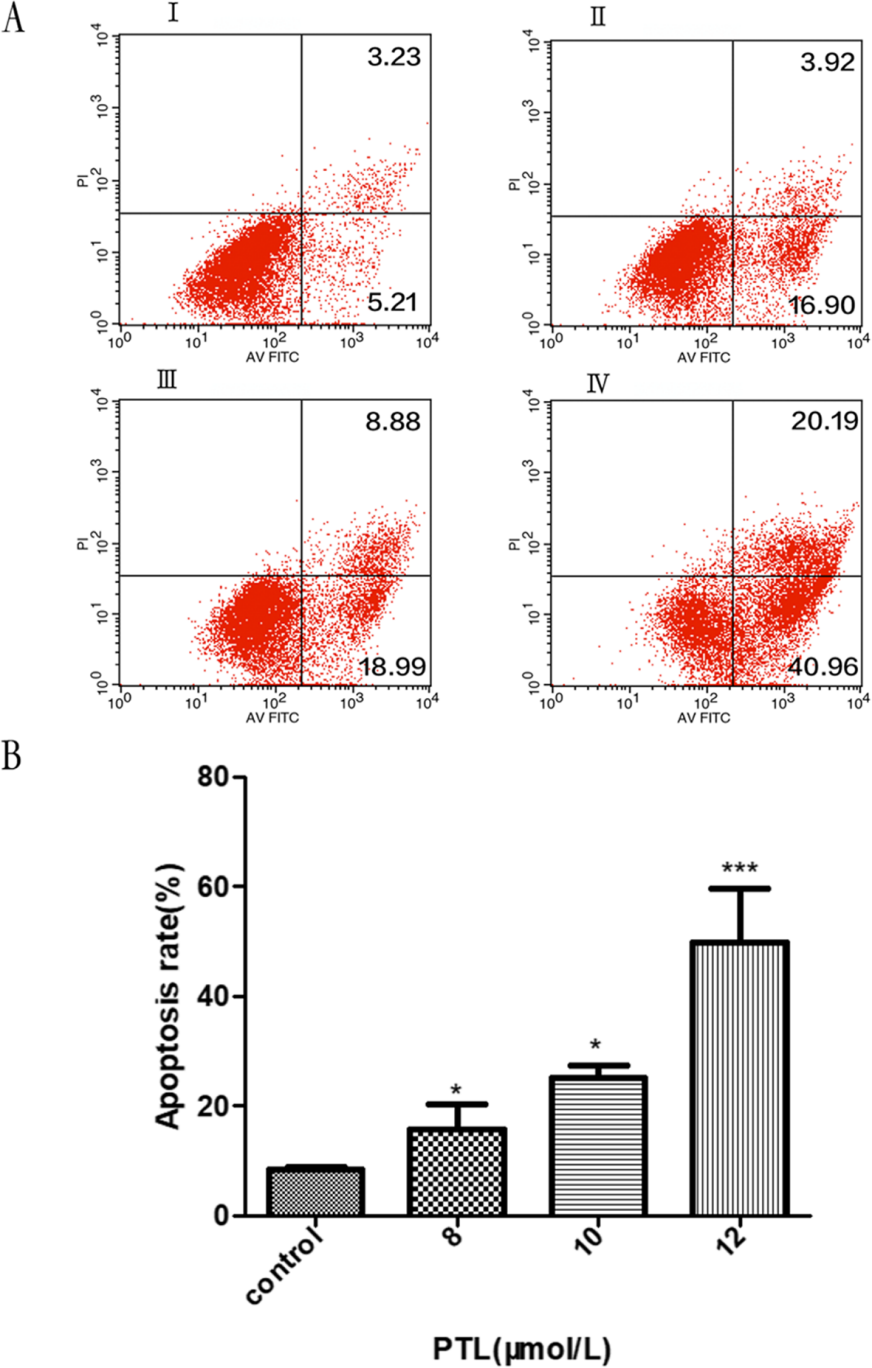
**A**: BCPAP Cell Apoptosis Rate were detected by Annexin V-FITC/PI Staining. I: control group; II: 8 μmol/L PTL group; III: 10 μmol/L PTL group; IV: 12 μmol/L PTL group. **B**: Statistical differences among the groups (*P < 0.05, ***P < 0.0001)

BCPAP cells were treated with different concentrations of PTL (8, 10, 12 μmol/L) for 24 h respectively. With the increase of PTL concentration, the apoptosis rate gradually increased, and the 12 μmol/L group was significantly higher than other groups. There were statistical differences among the groups (*P < 0.05 ***P < 0.0001) (Fig. 6).

### PRM verified the difference of apoptosis-related protein expression

**Fig. 7:**
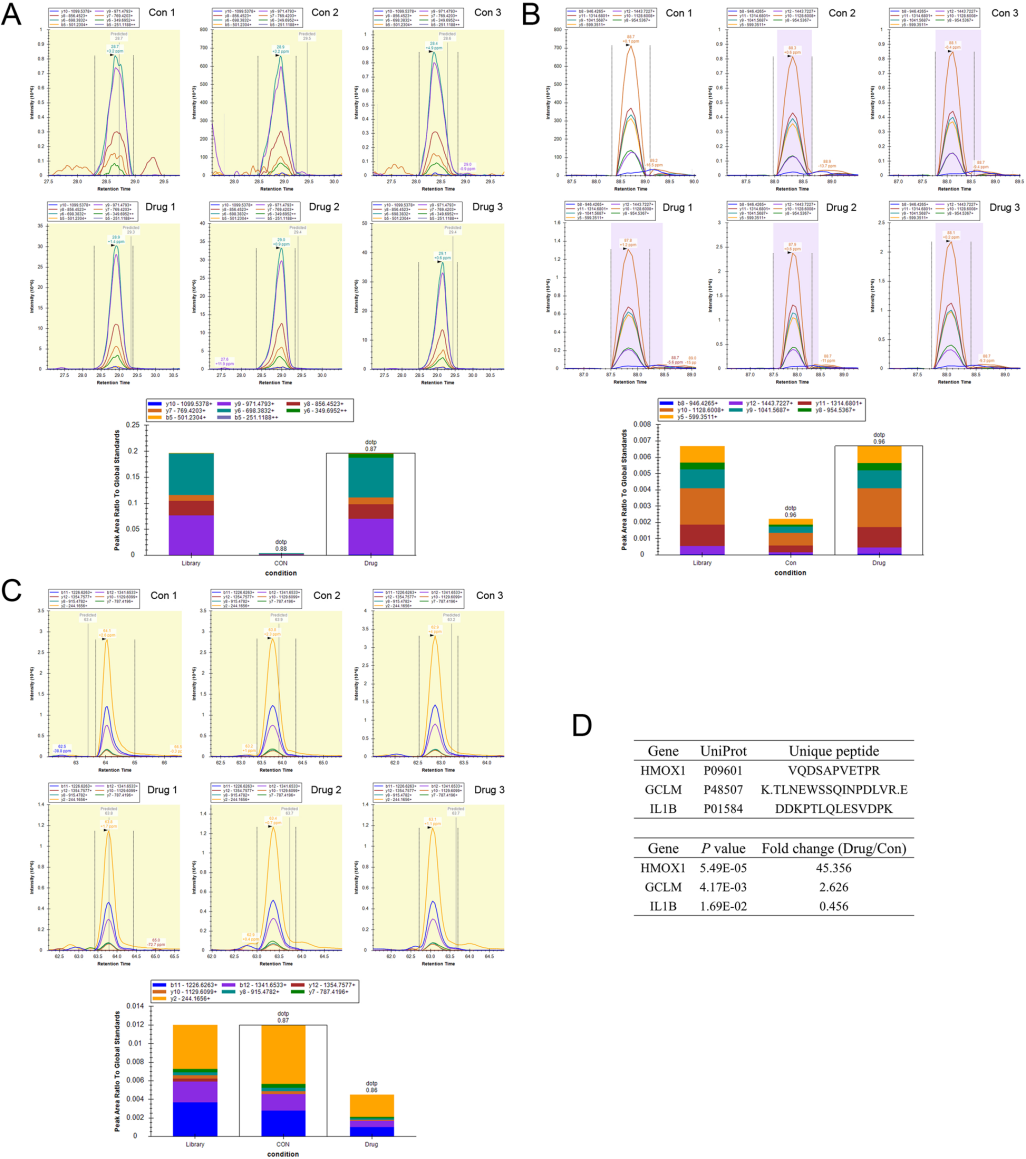
PRM analysis. **A**: HMOX1 protein expression increased in PTL treatment group; **B**: GCLM protein expression increased in PTL treatment group; **C**: IL1B protein expression decreased in PTL treatment group; **D**: Data and results of protein quantitative analysis

The above experiments confirmed that PTL can induce apoptosis of BCPAP cells, and in the proteomics clustering results, 6 proteins (IL1B, ITGA6, CASP8, GCLM, HMOX1 and HSPA1A) were classified into ‘negative regulation of extrinsic apoptotic signaling pathway’ (GO: 2001237). After bioinformatics screening, three proteins were selected to further verify expression differences by PRM. HMOX1 and GCLM were up-regulated and IL1B was down-regulated in BCPAP cells treated with PTL (Fig. 7).

### Quantitative real-time PCR verified the mRNA expression

**Fig. 8:**
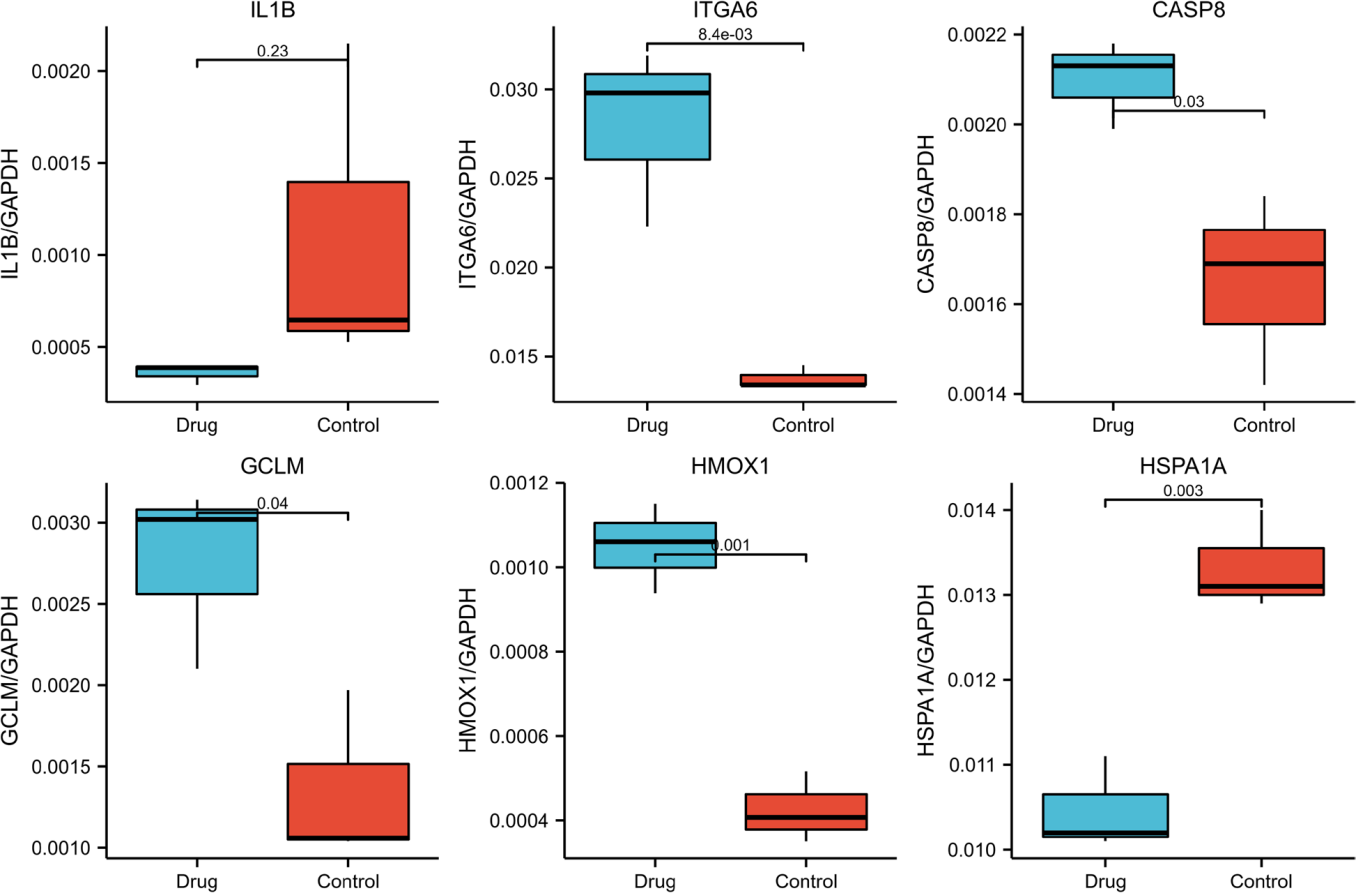
qRT-PCR results of IL1B, ITGA6, CASP8, GCLM, HMOX1 and HSPA1A mRNA expressions

PCR was used to verify the mRNA expression differences of the above-mentioned apoptosis-related genes. The mRNA expressions of HMOX1, GCLM, ITGA6 and CASP8 were up-regulated and HSPA1A was down-regulated. There was no difference in IL1B mRNA expression (Fig. 8).

## Discussion

Parthenolide is a sesquiterpene lactone, which is considered to be the main active ingredient in the anti-tumor effect of feverfew. As far as we know, the anti-tumor mechanism of PTL has not been fully elucidated. Previous studies have shown that PTL plays an anti-cancer role in prostate cancer, breast cancer, pancreatic cancer, oral cancer, colon cancer, glioblastoma multiforme and other tumors by inhibiting cell proliferation and inducing apoptosis [22–27]. Some previous studies suggested that PTL induces an anti-tumor effect, targeting nuclear factor-κB (NF-κB), producing reactive oxygen species and activating c-Jun N-terminal kinase [28–31]. Recent studies have shown that PTL inhibit the skeletal NF-κB signaling pathway to reduce prostate cancer related osteolysis [32]. A recent study have demonstrated that Dihydrotanshinone I, a tanshinone extracted from *Salvia miltiorrhiza Bunge*, can induce a decrement of NF-κB activity in anaplastic thyroid cancer paclitaxel-resistant cells SW1736-PTX and 8505C-PTX, resulting in decreased viability and clonogenic ability of the resistant cells [33]. Therefore, the effect of PTL on thyroid cancer may also be affected by targeting NF-κB pathway, further research is needed to confirm that. Recent studies have reported that PTL plays a role in inhibiting proliferation and promoting autophagy and apoptosis in thyroid cancer cells MDA-T32 [34]. Our previous studies also found that PTL can affect the energy metabolism and TCA cycle of thyroid cancer cells TPC-1, and the amino acid metabolism, choline metabolism and lipid metabolism of cells were changed after PTL treatment [35]. However, the cell line studied in that report is limited. So far, the proteomic differences between thyroid cancers treated with or without PTL have not been detailed reported. The underlying mechanism involved in PTL induced thyroid cancer cell death is not fully understood.

In recent years, high-throughput quantitative proteomics has become a widely used method to find disease-related factors [36–38]. In principle, the method of label-free quantification is applicable to any kind of samples, including those materials that are not easily labeled. Furthermore, unlike label-based methods, label-free method does not limit the number of samples to be compared. The present study is the first to explore the effect of PTL on thyroid cancer proteins using high-throughput quantitative proteomics analysis.

The results of this study showed that 156 proteins were significantly different in thyroid cancer treated with PTL and without PTL. In this study, GO and KEGG analyses were used to identify several significantly altered gene functions and signaling pathways. It is worth noting that a number of GO terms were related to cellular amino acid metabolic processes. This indicated that the anti-tumor effect of PTL may be related to changes in amino acid metabolism in cells. It is also important to note that there are 6 proteins (IL1B, ITGA6, CASP8, GCLM, HMOX1 and HSPA1A) classified into ‘negative regulation of extrinsic apoptotic signaling pathway’. PRM and PCR were used to verify the differential expression of these proteins and mRNA, PRM results of three proteins were consistent with proteomics results, but the PCR results were not entirely consistent. This may be due to the fact that mRNA expression and protein expression trends do not always coincide. It was not clear what role these differentially expressed proteins played in PTL-induced apoptosis, which might be the cause, the result or the compensatory reaction, which needs further study.

Apoptosis, also termed programmed cell death, is a way of maintaining normal tissue and organ function by removing damaged or abnormal cells. Apoptosis usually occurs in mature organisms, mainly in response to the stress that cells cannot resist through their own stress response mechanism, depriving cells of survival factors and promoting their programmed death [39]. In this study, after staining with Hoechst 33258, in the control group, there were many cells with high density, regular nuclear morphology, uniform and diffuse blue fluorescence. With the increase of drug concentration, the number of cells gradually decreased, cell morphology gradually irregular, cytoplasmic fluorescence gradually densification, loss of normal nuclear morphology. Annexin V-FITC/PI double-staining flow cytometry showed that the apoptosis rate of BCPAP cells increased under the treated of PTL. The aforementioned two experiments indicated that PTL can induce thyroid cancer cell BCPAP apoptosis in a concentration dependent manner.

With the rapid development of pharmaceutical technology, natural plant extracts gradually combine traditional medicine with modern medicine, and more and more scholars focus on the effects of natural drugs. As mentioned above, some studies have confirmed that PTL plays an anticancer or synergistic chemotherapy anticancer role in some tumors. Based on this, we conducted a preliminary cell experiment and found that PTL could inhibit the proliferation of papillary thyroid carcinoma. Therefore, proteomics analysis was performed and the differential proteins were clustered. The clustering results, such as amino acid metabolism, DNA replication and apoptosis, were closely related to the occurrence and development of tumors. Further cell experiments in this study also confirmed that PTL could promote apoptosis of thyroid cancer cells. Referring to the findings of other scholars, we believe that PTL has the potential to treat thyroid papillary carcinoma, and its molecular mechanism needs further study. At present, our team is conducting PTL experiments in animals, and the initial experimental results are in line with expectations.

## Conclusion

In summary, this is the first report applying the proteomic approach to identify proteins involved in the biochemical activity of PTL in thyroid cancer cells. Our study demonstrated that PTL exhibited an apoptosis-promoting function in BCPAP cells. The differential expressions of IL1B, ITGA6, CASP8, GCLM, HMOX1 and HSPA1A may contribute to the apoptotic in BCPAP cells under the treatment of PTL. This is worthy of further experimental exploration. Therefore, in terms of cell and protein expression, this study might provide a basis for further research on the mechanism of PTL and a potential direction for the clinical treatment of thyroid cancer.

## Data Availability

The datasets used and/or analysed during the current study are available from the corresponding author on reasonable request.

## Abbreviations

PTL: Parthenolide
IC50: 50% inhibitory concentration
RIPA: Radio immunoprecipitation assay
PMSF: Phenylmethanesulfonyl fluoride
RT: Room temperature
DEPs: Differentially expressed proteins
FDR: False discovery rate
NF-κB: Nuclear factor-κB
PRM: Parallel reaction monitoring
